# Chemical and Genetic Zebrafish Models to Define Mechanisms of and Treatments for Dopaminergic Neurodegeneration

**DOI:** 10.3390/ijms21175981

**Published:** 2020-08-20

**Authors:** Ola Wasel, Jennifer L. Freeman

**Affiliations:** School of Health Sciences, Purdue University, West Lafayette, IN 47907, USA; owasel@purdue.edu

**Keywords:** dj1, dopamine, MPTP, neurotransmission, paraquat, Parkinson’s disease, pink1, prkn, rotenone, zebrafish

## Abstract

The zebrafish (*Danio rerio*) is routinely used in biological studies as a vertebrate model system that provides unique strengths allowing applications in studies of neurodevelopmental and neurodegenerative diseases. One specific advantage is that the neurotransmitter systems are highly conserved throughout vertebrate evolution, including between zebrafish and humans. Disruption of the dopaminergic signaling pathway is linked to multiple neurological disorders. One of the most common is Parkinson’s disease, a neurodegenerative disease associated with the loss of dopaminergic neurons, among other neuropathological characteristics. In this review, the development of the zebrafish’s dopaminergic system, focusing on genetic control of the dopaminergic system, is detailed. Second, neurotoxicant models used to study dopaminergic neuronal loss, including 1-methyl-4-phenyl-1,2,3,6-tetrahydropyridine (MPTP), the pesticides paraquat and rotenone, and 6-hydroxydopamine (6-OHDA), are described. Next, zebrafish genetic knockdown models of *dj1*, *pink1*, and *prkn* established for investigating mechanisms of Parkinson’s disease are discussed. Chemical modulators of the dopaminergic system are also highlighted to showcase the applicability of the zebrafish to identify mechanisms and treatments for neurodegenerative diseases such as Parkinson’s disease associated with the dopaminergic system.

## 1. Introduction

The zebrafish (*Danio rerio*) is a well-established biomedical model that has been used in developmental toxicology, drug screening, genetics, and developmental and disease biology. The zebrafish genome has 70% homology with the human genome with 84% of zebrafish genes associated with human disease [1]. In addition, zebrafish can be easily maintained, have high fecundity, and develop relatively fast. Zebrafish embryos are transparent and develop externally, which allow for genetic manipulation of the embryos and monitoring of development ex vivo. Moreover, neurotransmitter systems are conserved between zebrafish and mammals, allowing translation of mechanisms of neurotransmission alterations and associated developmental and disease pathways [2]. All of these characteristics permit use of the zebrafish as an animal model to induce phenotypes observed in neurodegenerative diseases in humans. Successful models assist in understanding mechanisms behind neurodegenerative diseases and in identifying therapeutic targets.

Parkinson’s disease (PD) is a neurodegenerative disease characterized by motor and cognitive deficits. The motor impairment in PD is caused by the loss of dopaminergic (DA) neurons in the pars compacta in the substantia nigra. Both environmental and genetic factors contribute to the progression of PD, and multiple cellular and animal models have been developed for studying the mechanisms and therapeutic targets of PD, including zebrafish models [3].

The dopaminergic system in zebrafish is well characterized and is completely developed by 96 h post fertilization (hpf) [4]. DA neuronal populations in zebrafish larvae that resemble the human substantia nigra have been identified, supporting the zebrafish as a promising model for induction of the PD phenotype and allowing for high-throughput analysis for screening therapeutic drugs. In this review, dopamine signaling and development of the DA system in zebrafish are detailed to understand the signaling pathway and the roles of different genes in comparison to mammals. Next, the use of chemical exposure to generate zebrafish models of DA neuronal loss, including 1-methyl-4-phenyl-1,2,3,6-tetrahydropyridine (MPTP), the pesticides paraquat and rotenone, and 6-hydroxydopamine (6-OHDA), is described. Zebrafish knockdown models of key PD genetic targets are also discussed. Lastly, chemical modulators of the zebrafish DA system are highlighted to support the applicability of the zebrafish to identifying mechanisms of and treatments for dopaminergic neurodegeneration.

## 2. Dopamine Signaling in Zebrafish

**Figure 1:**
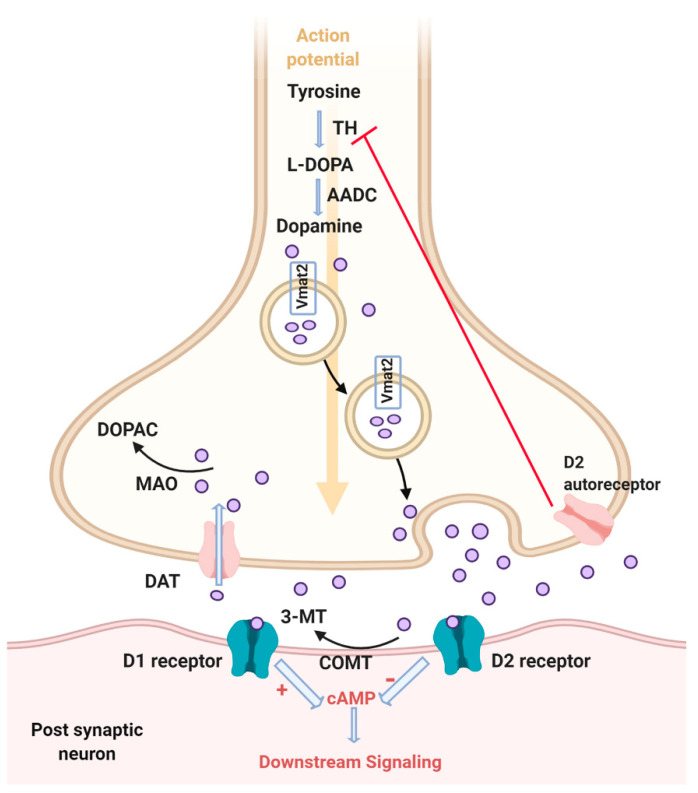
Dopamine signaling pathway. Tyrosine is converted to L-DOPA by the rate limiting step enzyme, tyrosine hydroxylase (TH). Then dopamine is synthesized by action of the aromatic amino acid decarboxylase (AADC). Dopamine is packed in the cytosol via vesicular monoamine transporter 2 (Vmat2). Dopamine is then released from vesicles into the synaptic cleft in response to an action potential. Dopamine can either bind to D1 receptor and activate adenylate cyclase and consequently activate downstream signaling through cAMP, or bind D2 receptor and inhibit adenylate cyclase and downstream signaling. Reuptake of dopamine from the synaptic cleft to the cytosol occurs through the dopamine transporter (DAT). Then dopamine can either be degraded to 3,4-dihydroxyphenylacetic acid (DOPAC) via monoamine oxidase (MAO) or repacked in vesicles via Vmat2. Dopamine can also be degraded in the synaptic cleft via catechol-O-methyltransferase (COMT) to 3 methyltyramine (3MT). Extracellular dopamine can bind to the D2 autoreceptor, which inhibits the synthesis of dopamine. Created with BioRender.com.

Dopamine is a catecholamine neurotransmitter synthesized by oxidation of the amino acid tyrosine. Tyrosine hydroxylase (TH) converts tyrosine to dihydroxyphenylalanine (L-DOPA), which is converted to dopamine by the action of aromatic amino acid decarboxylase (AADC) (Figure 1). Dopamine is a precursor of other catecholamine neurotransmitters, noradrenaline, and adrenaline. Oxidation of dopamine with dopamine β hydroxylase results in formation of noradrenaline. There are five families of dopaminergic receptors. Those receptors are G-protein coupled receptors and are classified into two types according to their effects on adenylate cyclase. D1-like receptors (D1 and D5) activate adenylate cyclase and the downstream targets, whereas D2-like receptors (D2, D3, and D4) inhibit adenylate cyclase and the downstream targets [5]. In the central nervous system, dopamine is transported from the cytoplasm into secretory vesicles by vesicular monoamine transporter (VMAT2) (also known as solute carrier family 18 member 2 (SLC18A2) (Figure 1)) [6]. Dopamine uptake from the synapse occurs by presynaptic dopamine transporters called solute carrier family 6 member 2 (SLC6A2) (also called dopamine transporter (DAT)). SLC6A2 transports dopamine from the synaptic cleft back to the cytosol, which is influenced by an ion concentration gradient generated by the plasma membrane Na^+^/K^+^ ATPase [7]. Then, dopamine can be packed in the synaptic vesicles or metabolized. Dopamine is metabolized by monoamine oxidase (MAO) in the cytosol into 3,4-dihydroxyphenylacetic acid (DOPAC) (Figure 1). Mammals have two isozymes, MAO-A and MAO-B. MAO in zebrafish is similar to MAO-A [8]. MAO-B is mainly responsible for degradation of serotonin. In mammals, dopamine can be degraded in the synaptic cleft by catechol-O-methyltransferase (COMT) into 3 methyltyramine (3MT) and converted to homovanillic acid by MAO. There are two forms of *COMT* genes in zebrafish, *comta* and *comtb*. The role of COMT in metabolizing dopamine in zebrafish is not well understood, but Sallinen et al. (2009) suggested that the presence of 3MT, the product of dopamine methylation, is evidence of the role of COMT in metabolizing dopamine. Another study showed that *comta* mRNA expression was abundant in the gut, gills, and spleen, while *comtb* mRNA expression was abundant in the liver and brain, highlighting that *comtb* is more relevant to central nervous system than *comta* [9]. Although COMT inhibition did not result in an increased level of dopamine in larval zebrafish, an increase in DOPAC was observed, indicating activation of oxidative metabolism [9]. D2 autoreceptor plays a role in inhibiting neurotransmission by a negative feedback mechanism. Horzmann and Freeman (2018) discussed in detail the comparison between genes involved in catecholamine neurotransmission in zebrafish and human genes [10]. Neurons that express *tyrosine hydroxylase* (*th)* and do not express *β hydroxylase* are considered dopaminergic neurons [11]. Holzeschuh et al. (2001) suggested that *dopamine transporter* (*dat)* expression distinguishes dopaminergic neurons from noradrenergic and adrenergic neurons [12]. This study showed that neurons that express *th* and do not express *dat* are adrenergic and noraderengic and are found in the locus coeruleus and medulla. It was proposed that zebrafish have another TH-encoding gene, *th2*, but it was found that this gene encodes for tryptophan hydroxylase and should be used as a marker for serotonin neurons [13].

## 3. Distribution of Dopaminergic Neurons in the Zebrafish Brain

Catecholaminergic (CA) populations in zebrafish larvae were determined by several groups, but different nomenclature were used to identify them [14]. Rink and Wullimann (2002) classified CA populations in different brain areas in larval zebrafish [4]. The telencephalon contains CA populations in the olfactory bulb (OB) and subpallium (SP). According to this study, there were eight CA populations in the diencephalon, DC1–DC8. The rhombencephalon has CA in the preoptic area (PO), locus coeruleus (LC), medulla oblongata (MO), and area postrema (AP). Sallinen et al. (2009) classified CA populations into 17 populations (populations 1–17). The midbrain of zebrafish does not have any DA neurons, in contrast to the mammalian brain. It has been found that larval DA neurons in DC1, DC2, and DC4 (ventral thalamic and posterior tuberculum in adult brain) send projections to the subpallium in adult zebrafish. This pathway is hypothesized to be homologous to DA neurons in the substantia nigra and ventral tegmental area (DA groups A9 and A10, respectively) that project to the striatum in the mammalian brain [15]. Du et al. (2016) found that axons from the periventricular nucleus of the posterior tuberculum projected to the subpallium in 120 hpf larvae [16]. Lam et al. (2005) observed that an embryonic exposure to 1-methyl-4-phenyl-1,2,3,6-tetrahydropyridine (MPTP) decreased the number of DA neurons in the diencephalon in zebrafish embryos. Additionally, 48 hpf embryos treated with MPTP showed a weak reflex in response to touch stimuli compared to control fish. In addition, the decrease in DA neurons in the diencephalon is reversed in MPTP-treated embryos by deprenyl, a monoamine oxidase β inhibitor that inhibits conversion of MPTP to its metabolite MPP^+^ (1-methyl-4-phenylpyridinium) [17]. Sallinen et al. (2009) showed that populations 5, 6, and 11 (DC2 and DC4, according to Rink and Wullimann’s nomenclature) in 120 hpf zebrafish larvae were sensitive to MPTP and MPP^+^, which induced a transient decrease in tyrosine hydroxylase-positive cells and a decrease in swimming activity [18]. Those studies confirmed that populations 5, 6, and 11 (preoptic, thalamic, and posterior tuberculum DA neurons in the adult zebrafish brain) in the diencephalon represent the mammalian midbrain DA neurons that are sensitive to MPTP, and the loss of DA neurons caused motor deficits. Another study suggested that DA clusters DC2 and DC4 in zebrafish larva (periventricular posterior tuberculum and posterior tuberculum in adult brain) correspond to DA in the caudal diencephalon of mammals (A11) [19]. This study showed that differentiation of DA neurons in the periventricular posterior tuberculum and posterior tuberculum was dependent on the transcription factor orthopedia (*otp*) and sent ascending projections to the ventral diencephalon and subpallium, and descending projections to the pretectum, tectum, thalamus, hypothalamus, hindbrain, and spinal cord. Mammalian population A11 is also dependent on *OTP* expression and sends ascending neocortical and descending diencephalospinal projections [20]. Lambert et al. (2012) found that the dopaminergic otp-dependent neurons mediate the development of locomotor activity via spinal dopamine receptor D4 (D_4_R) signaling [10]. This study is in agreement with Thirumalai et al. (2008) who saw differential effects of dopamine during development. They reported that dopamine suppressed spontaneous swimming via D2 receptors at 72 hpf, but this inhibition of activity did not occur in 120 hpf larvae [10]. It was observed that the number of neurons in the periventricular of posterior tuberculum increased from 24 to 72 hpf and then decreased from 72 to 120 hpf [16].

## 4. Genetic Control of Dopaminergic System Development in the Zebrafish

**Figure 2:**
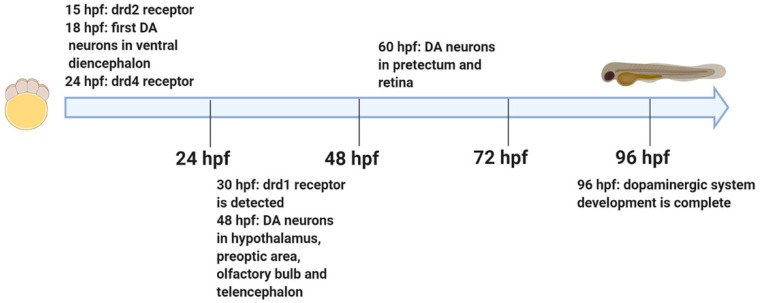
Developmental timeline of the dopaminergic system in zebrafish. DA: dopamine; drd1: dopamine receptor d1; drd2: dopamine receptor d2; drd4: dopamine receptor d4; hpf: hours post fertilization. Created with BioRender.com.

Understanding the timeline, signaling pathways, and molecular factors that control the development of different population of the DA system is important to establishing disease models and also assessing the abnormalities that can be induced by toxins and toxicants. As mentioned earlier, DA neurons are characterized by expressing *th* and *dat*. The first DA neurons can be detected in the ventral diencephalon at 18 hpf (Figure 2) [12]. D2 and D4 receptors can first be detected at 15 hpf and 24 hpf, respectively [21,22]. D1 receptor gene (*drd1*) is expressed at 30 hpf in the developing diencephalon and hindbrain (Figure 2) [23]. DA neurons are first detected in the olfactory bulb, preoptic area, telencephalon, and hypothalamus at 48 hpf (Figure 2) [4,12]. DA neurons are observed in the pretectum and retina at 60 hpf (Figure 2) [4,12]. By 96 hpf, all dopaminergic neurons are formed (Figure 2) [4]. Detailed information on early development of DA neurons in zebrafish was published previously by Rink and Wullimann (2002) [4] and Holzschuh et al. (2001) [12].

Mutant models in zebrafish allow studying of the signaling pathways that affect the development of the DA system. In contrast to mammals, zebrafish embryos that have a mutation may survive, as early development depends on maternal proteins stored in yolk and/or two copies of a mammalian gene may be present in the zebrafish genome [24]. Development of different DA populations is affected by different signaling pathways [25]. The pretectal and retinal amacrine DA neurons require sonic hedgehog (shh) signaling as mutant shh (*syu*) and co-receptor, when smoothed, reduce the number of those cells, while early ventral diencephalic and olfactory bulb DA populations form normally. It was suggested that Shh affected the precursors of DA neurons in the pretectum. In mutant *ace*, which lacks *fgf8*, early DA neurons in the posterior tuberculum developed normally, but lack of DA in the anterior ventral diencephalon was observed at 72 hpf. On the other hand, the ventral diencephalon required the nodal pathway to develop. Mutations in *cyc*, which affects Nodal related protein 2, or *oep,* which is essential for Nodal and receptor interactions, caused complete deletion of Nodal signaling leading to a significant decrease in ventral diencephalon neurons [25]. Therefore, it is unclear whether the absence of DA neurons was directly caused by defects in specification of DA neurons in the ventral diencephalon or because of defects in patterning [25].

The roles of transcription factors in the development of DA neurons have been studied. Expression of nuclear receptor subfamily 4, group A member2 (*nr4a2*) was found in DA neurons in the preoptic area, pretectum, and retinal amacrine and was suggested to be required in specifications of these cells (Table 1). Combined morpholino antisense oligo-mediated knockdown of *nr4a2a* highlighted that Nra4a is required for *th* and *dat* expression in the preoptic area, pretectum, and retinal amacrine [26]. Expression of *nr4a2* was found in cells expressing *elavl3*, a marker of postmitotic differentiating neurons, and not in *sox2* expressing stem and progenitor cells, indicating that *nr4a2* is required for late aspects of DA differentiation [26]. In contrast to mammals, *nr4a2a* was not expressed in DA neurons in the posterior tuberculum and ventral thalamus, suggesting *nr4a2a* was not required for differentiation of DA neurons that send ascending projections [26]. On the hand, Luo et al. (2008) found that *nr4a2b* did not co-express with *th* and *dat* in the posterior tuberculum, but knockdown of Nr4a2 decreased expression of *th* and *dat* expressing DA neurons in the posterior tuberculum [27]. Furthermore, a decrease in dopamine was observed and this decrease was reversed by injecting mouse *Nr4a2* mRNA. It is important to note that Luo et al. (2008) used a higher concentration of morpholino compared to the previous study [27].

Neurogenin (*ngn1*) is required in the specification of DA progenitor cells. In absence of *ngn1* activity, progenitor cells were formed, but failed to differentiate to DA neurons. In addition, supernumerary DA neurons formed in the forebrain of zebrafish when *ngn1* was overexpressed [28]. This same study found that FEZ family zinc finger 2 (*fezf2*)/FEZ family zinc finger 1 (*Fezf1*) is responsible for regulation of *ngn1*-expressing DA precursors in the diencephalon [28].

Transcription factors paired-like homeodomain transcription factor 3 (Pitx3) and LIM homeobox transcription factor 1 (Lmx1b) are essential for mammalian mesencephalic specification; thus, their role in zebrafish was investigated [26]. Knockdown of *pitx3* did not cause any abnormality in DA neuronal development. *lmx1b* was expressed in a diencephalon region that contains precursor cells for DA neurons, and it was not expressed in cells that expressed *th*. Although *lmx1b* was not expressed in DA neurons, the knockdown of both *lmx1b* paralogs, *lmbx1bb* and *lmx1ba* (also known as *lmx1b.1* and *lmx1b.2*), caused a decrease in the number of diencephalic DA neurons that sent ascending projections (Table 1). In addition, knockdown expression of *lmx1b* affects expression of *pitx3* and *nr4a2* genes in the ventral diencephalon, suggesting that *lmx1b* expression is required for differentiation or maintenance of DA neurons. The authors of this study suggested that these observations may provide a molecular explanation for the shift of ascending DA neurons from the diencephalon to mesencephalon during vertebrate evolution. They hypothesized that rostrocaudal and dorsoventral patterning established a precursor domain that extends from the diencephalon to ventral mesencephalon. This precursor domain is characterized by co-expression of *lmx1b* and *pitx3* in the posterior tuberculum and hypothalamus. In addition, the expression of *lmx1b* was found in the midbrains of zebrafish.

The role of Aristaless related homeobox (Arx) and islet1 (Isl1) in the development of DA neurons was studied by induction of *arx* and *isl1* knockdown via antisense morpholino microinjections in morphant larvae (Table 1) [29]. Results of this study showed that inhibiting expression of *arx* and *isl1* caused a decrease in expression of *th* in prethalamic DA neurons. The inhibition of Arx activity led to improper dorso/ventral patterning of the prethalmus and loss of prethalamic DA, whereas Isl1 inhibition did not affect the morphology of the diencephalon nor change expression of the other transcription factors. Knockdown of *isl1* lead to a reduction of *th* and *dat* expression in the prethalamic domain. The authors of the study suggested that Isl1 can be required for the differentiation of prethalamic DA neurons.

As mentioned earlier, *otp* is required for development of DA neurons in the posterior tuberculum and hypothalamus (Table 1). Giacco et al. (2006) showed that Nodal signaling regulated the *otp* expression that is required for differentiation of CA neurons in the posterior tuberculum [30].

The loss of function of glial cell line-derived neurotrophic factor (*gdnf*) led to alterations in expression of multiple transcription factors that are involved in DA neuron development in zebrafish, including *lmx1b, otp*, and *nr4a2* (Table 1) [31]. Knockout of *gdnf* decreased expression of *otpb* in the preoptic region and posterior tuberculum at 72 hpf. Loss of *gdnf* led to the absence of *lmx1b* expression in the posterior tuberculum. Loss of expression of *gdnf* caused a decrease in expression of *nr4a2* only in the hindbrain, but not in the diencephalon. Expression of *pitx3* did not change with the loss of *gdnf* expression, which is consistent with previous studies that showed that differentiation of DA neurons in the posterior tuberculum is not dependent on *pitx3*.

It was demonstrated that the majority of DA neurons in zebrafish can synthesize gamma-aminobutyric acid (GABA), which was proven by co-expression of *th* and *glutamate decarboxylase 1b/2* (*gad1b/2*) markers for GABAergic neurons [32]. DA neurons that express *gad1b/2* were located in areas that have GABAergic neurons, such as the preoptic region, subpallium, and thalamus. DA neurons that do not express *gad1b/1,* express vesicular glutamate transporter 2 *(vglut2a/b),* a marker for glutaminergic neurons [32]. DA neurons that express glutaminergic markers are the *otp*-dependant neurons in the posterior tuberculum [32]. Since *ngn1* is expressed in glutaminergic neurons in the posterior tuberculum, but not in the hypothalamus, it was suggested that *ngn1* activity affects only DA neurons expressing *vglut2a/b* and not DA neurons expressing *gad1b/2* in the hypothalamus [32].

## 5. Dopaminergic Neurotoxicant Models in Zebrafish

### 5.1. 1-Methyl-4-Phenyl-1,2,3,6-Tetrahydropyridine (MPTP)

MPTP is a neurotoxicant that targets DA cells and is used to induce loss of DA neurons to establish PD models in animals [33]. MPTP is lipophilic and can readily cross the blood–brain barrier (BBB). Toxicity of MPTP is caused by its metabolite, 1-methyl-4-phenylpyridinium ion (MPP^+^), that is formed by MAO-B in astrocytes. MPP^+^ is transported to DA neurons via DAT. MPP^+^ inhibits mitochondrial complex 1, causing an increase in reactive oxygen species (ROS) and eventually cell death [34], which causes motor dysfunction similar to that observed in PD patients.

Reduction in DA neurons and behavioral changes following exposure to MPTP were assessed in zebrafish in many studies [17,18,35,36]. Larval DA neurons in the pretectum region, ventral diencephalon, olfactory bulb, and hypothalamus are sensitive to MPTP treatment [17,18,35,36], but no effect on DA neurons in the telencephalon was observed [36]. Furthermore, transgenic zebrafish with enhanced green fluorescent protein (EGFP)-labelled monoaminergic neurons, where the expression of GFP is controlled by the expression of *vmat2*, Tg(vmat2:EGFP), were used to assess the neurotoxicity of MPTP. It was found that *th*^+^ neurons in the posterior tuberculum in the ventral diencephalon were more sensitive to MPTP [37]. Furthermore, a transgenic model that expresses EGFP under control of *dat,* a more DA-specific marker, Tg(dat:EGFP), showed a significant loss of ventral diencephalon DA neurons after two days of MPTP exposure [38].

A study found that exposure to 45 mg/L MPTP from 24 to 120 hpf caused a decrease in diencephalic DA neurons at 120 hpf, which was assessed by *th* in situ hybridization [35]. Additionally, a decrease in swimming speed with 9 or 45 mg/L MPTP was observed. A reduction in DA neurons in the pretectum and ventral diencephalon at 96 hpf was observed using in situ hybridization of *dat* and *th* with relatively low concentrations of 5 and 10 µg/mL MPTP [36]. This study confirmed that MPTP’s mechanism of toxicity is similar to what it is in other mammalian models. Deprenyl, MAO inhibitor or DAT inhibitor, and nomifensine treatments protected neurons from MPTP effects, confirming the role of MAO and DAT in the reduction of DA neurons [36]. In addition, DAT knockdown decreased neuronal loss in ventral diencephalon caused by 5 µg/mL and reversed the slow response to touch, although inability to swim away was not reversed, indicating that DA neurons may mediate response to touch movement [36]. Reduction of DA neuron loss caused by deprenyl was observed in other studies [17,18]. Sallinen et al. (2009) found that exposure to 1000 µM from 1 to 4 hpf caused a decrease in total distance moved at 120 and 144 hpf, but not at 168 hpf. Additionally, the decrease in TH^+^ cells in the pretectum, diencephalon, preoptic region, and hypothalamus was only observed at 120 hpf, but not at 168 hpf, indicating that the effect MPTP is transient [18]. Exposure to MPTP from 72 to 120 hpf showed a decrease in TH in pretectum DA neurons along with a decreased number of movements initiated by 120 hpf, while no changes were observed in the diencephalon [39].

It is important to highlight that these previously mentioned studies had different exposure regimes, including age at start of exposure, age of assessment, length of exposure, concentrations of MPTP, and use of different markers to characterize DA neurons. These factors highly affect the results and their interpretations. For example, exposures that started at 72 hpf when the BBB was formed would explain different results compared to an exposure that started at 24 hpf, when DA neurons were developing and the BBB was immature.

MPTP treatment in adult zebrafish caused a decrease in total distance moved and mean velocity and an increase of freezing bouts [35,40,41]. However, a decrease in total distance moved and mean velocity was transient as it was no longer observed at nine days after exposure to MPTP [41]. Neither changes in number of TH^+^ cells in MPTP-treated adult zebrafish [35,40], nor signs of neuronal death were observed [41]. Unsurprisingly, treatment of adult zebrafish with MPP^+^ did not induce alterations in locomotor activity or DA neurons [35]. This result can be explained by the inability of MPP^+^ to cross the BBB.

### 5.2. Paraquat and Rotenone

Exposures to the pesticides paraquat and rotenone have been linked to development of parkinsonism. Paraquat is a herbicide with a chemical structure similar to MPP^+^ that cannot readily cross the BBB [42]. Rotenone is lipophilic and can cross the BBB [33]. Rotenone and paraquat induced PD-like symptoms in rodent models. [43] On the other hand, contradicting results have been obtained in paraquat-treated zebrafish. One study found that exposure in larval zebrafish to 10 mg/L of paraquat from 24 to 120 hpf did not result in significant changes in number of TH^+^ cells or changes in locomotor activities [35]. Another study showed that exposure to 0.04 mg/L of paraquat at 18 to 96 hpf caused a 60% reduction in DA neurons and a 50% reduction in serotonergic neurons, in addition to an increase in oxidative stress [44]. It is important to note that 0.04 mg/L was the observed LC50 in this study, which suggests that the observed effect may be due to lethality or malformation of the embryos and not directly caused by paraquat. Another study found that exposure to 100 mg/L at 72 hpf caused activation of oxidative stress related genes after 24 h of treatment [45]. A more recent study showed that transgenic zebrafish Tg(dat:EGFP) treated with 257 mg/L of paraquat had decreased expression of *th* and *dat* and a reduction in total DA neurons with a reduction in overall decrease of fish size [46]. From these studies, it is sensible to conclude that only high concentrations that lead to developmental abnormalities or complete mortality are able to induce neurodegeneration in zebrafish.

Paraquat exposure in adult zebrafish provides contradicting results. Repeated intraperitoneal injection of paraquat caused an increase in dopamine level and a decrease in DOPAC levels, while no changes in TH levels were observed at 16 days after exposure [47]. Additionally, this study showed that paraquat-treated fish had lower motor activity, but no change in anxiety-related behavior [47]. On the other hand, another study showed that repeated intraperitoneal exposure caused an increase in aggressive behavior and non-motor patterns related to defensive behaviors [48]. Although increases in antioxidant enzyme activities and a decrease in mitochondrial viability were observed, no changes in reactive oxygen species levels occurred [48]. These effects are similar to what was observed in rodents models [49,50]. As reviewed in Vaz et al. (2018) paraquat-rodent models also showed some variability in neurotoxic effects [51].

Rotenone exposure also results in conflicting findings. A study showed that exposure to 5 or 10 µg/L from 24 to 120 hpf caused no changes in locomotor activities or changes in DA neurons [35]. Similar effects were observed in adults treated with 2 µg/L for 4 weeks [35]. A recent study showed exposure to 50 nM rotenone starting at 72 hpf for four days caused loss of DA neurons in the ventral diencephalon, in addition to decreased locomotor activity [46]. In this study, upregulation of *dat* expression was observed. The authors suggested that this increase in *dat* expression may be caused by a compensatory mechanism to increase dopamine uptake in surviving neurons [46]. Although this study showed that rotenone can induce some of the PD phenotype, it also caused cardiac edemas [46]. The differences in the observed effects in these different studies may indicate the role of rotenone toxicity in the development and survival of DA neurons.

### 5.3. 6-Hydroxydopamine (6-OHDA)

6-OHDA has a chemical structure that is close to those of catecholamines; therefore, 6-OHDA has affinity to the dopamine transporter and norepinephrine transporters [43]. 6-OHDA is hydroxylated dopamine, and because of its polarity, 6-OHDA cannot cross the BBB. Feng et al. (2014) found that treatment of larvae at 48 to 96 hpf with 250 µM 6-OHDA caused a decrease in locomotor activity at 168 hpf. The authors found that the defects in locomotor activities were reversed by adding vitamin E (antioxidant) and minocycline (microglia inhibitor), indicating that oxidative stress and inflammation are involved in 6-OHDA’s mechanism of toxicity [52]. Interestingly, a more recent study showed that exposure to 1 µM 6-OHDA at 72 hpf caused a significant decrease in the number of DA neurons, although no change in locomotor activity was observed [52]. In addition, larvae developed severe cardiac malformation after exposure to 1 µM 6-OHDA.

Intramuscular injection of 6-OHDA in adult zebrafish caused a decrease in locomotor activity at six days after the injection, but this effect was not observed at nine days post injection. This behavioral change was accompanied with a decrease in dopamine and noradrenaline levels, although no change in TH level was observed. To develop a model that mimics the loss of DA neurons in the substantia nigra in PD, injection of 6-OHDA directly into the ventral diencephalon was done and led to the loss of more than 85% of the DA neurons in the preoptic area, posterior tuberculum, and hypothalamus [53] and a decrease in locomotor activity. The difference between effects caused by intramuscular and direct injection into the diencephalon can be explained by the inability of 6-OHDA to cross the BBB. The decrease in locomotor activity after intracranial injection of 6-OHDA was recovered after 30 days post exposure [53]. The authors suggested that this model can be useful for investigating the neuronal regeneration ability of adult zebrafish [53].

## 6. Genetic Zebrafish Models of Dopaminergic Neurodegeneration

As mentioned above, PD is characterized by loss of DA neurons in the substantia nigra in the human brain. It was thought that this cell death is caused by a combination of factors called the “multiple-hit” hypothesis, which suggests that genetic factors and environmental factors contribute to the development of PD [3]. As reviewed in Sager el al. (2010), multiple genes that were linked with PD development are found in zebrafish such as those that encode for Dj-1, Pink1, Parkin, and Lrrk2 [54]. According to the two-hit theory, cell death can happen due to dopamine oxidation or mitochondrial dysfunction accompanied inhibition of the protective effect caused by loss of function of Parkin [3].

Using morpholino antisense oligonucleotides, transient knockdowns of PD-associated genes were induced to understand their roles in the zebrafish. Genetic models using knockdowns of zebrafish *dj-1*, *pink1*, and *prkn* were made to establish zebrafish PD models.

Mutations of *DJ-1* are linked with early onset PD. DJ-1 has a role in protection against oxidative stress [54]. Knockdown of *dj-1* in zebrafish larvae caused an increase in p53 and BAX expression levels without induction of any changes on neuronal cells. Exposure to hydrogen peroxide and the proteasome inhibitor MG132 to *dj-1* deficient zebrafish caused loss of DA neurons with an increase in p53 expression. Neuronal cell death in DJ-1 deficient mice was observed after treatment with MPTP and amphetamine [55]. Interestingly, inhibition of p53 before or after MG132 prevented neuronal death. Moreover, neuronal cell death was observed in zebrafish with knockdown of *dj-1* and *mdm2*, a negative p53 regulator, even without adding xenobiotics. Overall, these data confirm that p53 mediates neuronal cell loss caused by DJ-1 deficiency [56].

PTEN (phosphatase/tensin homolog)-induced putative kinase (PINK1) mutation is linked with early-onset PD, although its function is unknown. Xi et al. (2010) showed that morpholino oligonucleotide mediated knockdown of *pink1* caused alterations in patterning of DA neurons in the ventral diencephalon and their projections without changing their number [56]. Additionally, reduced response to tactile stimuli and reduced swimming was observed. These behavioral changes were recovered by treatment of exogenous *pink1*. Additionally, treatment with SKF-38393, a dopamine receptor agonist, rescued larval response to touch. The authors suggested that these data indicate the ability of larvae to respond to dopaminergic stimulus, although the improper patterning lead to hypoactivity due to improper projections towards the forebrain [57]. Another study showed that inactivated translation of *pink1* by morpholino-oligonucleotides in zebrafish embryos decreased *th* expression at 120 hpf, while no change in *dat* was observed. This study showed that lack of *pink1* expression increased sensitivity to MPTP that lead to locomotor deficiency and loss of some populations of DA neurons in ventral diencephalon [58]. This result highlights the role of genetic factors in mediating oxidative stress-induced PD.

Autosomal recessive inherited mutations in the gene encoding the E3 ubiquitin ligase, Parkin are the most common cause of early onset PD [59]. Knockdown *prkn* in zebrafish embryos caused a decrease in *th*^+^ expressing neurons in the diencephalon and a decrease in mitochondrial complex 1 activity, while no change in locomotor activity was observed. As explained by the authors, an inhibition in mitochondrial complex 1 and loss of DA neurons are similar effects that are observed in humans with *PRKN* mutations, confirming the suitability of zebrafish to be used in drug screening and investigating mechanisms behind neuronal cell death in early onset PD [60]. Furthermore, exposure of *prkn* knockdown zebrafish embryos to MPP^+^ caused a significant decrease in the DA diencephalic neurons compared to unexposed embryos, indicating a synergistic effect between MPP^+^ and *prkn* knockdown.

It should be noted that although morpholino mediated knockdown is a helpful tool to inhibit expression of genes, other techniques such as Clustered Regularly Interspaced Short Palindromic Repeats/CRISPR-associated protein-9 nuclease (CRISPR/Cas9) that are now commonly used in biological research will help to avoid off-target inhibition or ineffective inhibition and will increase reliability and reproducibility of future studies with zebrafish PD genetic models.

## 7. Chemical Modulators of the Dopaminergic System in Zebrafish

Clinical and experimental drugs have been used to modify different targets in the dopamine signaling pathway using the zebrafish. Modulation of dopamine signaling is useful to validate proposed disease models, identify potential therapeutic targets, and also identify phenotypes related to changes in dopamine signaling due to exposure to environmental chemicals. 

A study was performed to compare the effects of dopamine receptor agonists and antagonists on zebrafish to their effects in mammals [61]. Larval zebrafish exposed to DA agonist, SKF-38393 (D1 agonist), quinpirole (D2-agonist), or apomorphine at 144 hpf showed hyperactivity in the light/dark locomotor activity test, while exposure to DA antagonists, haloperidol (D2-antagonist), SCH-23,390 (D1-antagonist), or butaclamol caused a decrease in locomotor activity. Selective receptor agonists (SKF-38393, quinpirole) and selective antagonists (haloperidol, SCH-23,390) showed a dose dependent decrease in locomotor activity, while non-selective receptor agonists and antagonists caused a biphasic behavioral pattern. These results showed that zebrafish exposure to receptor agonists and antagonists causes similar effects as in mammals, allowing for the use of these drugs on zebrafish to predict phenotypic changes in mammals after exposure to environmental toxicants.

Exposure to the dopamine receptor antagonists, haloperidol or chlorpromazine, caused a decrease in the mean velocity resulting in a reduction in the frequency of initiation of spontaneous movements [62], indicating the role of dopamine in initiating the movement. Exposure to the dopamine receptor agonists, apomorphine and ropinirole, caused an increase in active velocity, which is defined as displacement per time spent moving, in treated larvae.

Embryonic exposure to D1 receptor antagonist SCH-23,390 or the D2 receptor antagonist haloperidol caused a decrease in locomotor activity, as assessed in the light/dark locomotor assay [63]. Differential effects were observed between the two zebrafish strains that were used in this study, AB and 5D [63]. While exposure to SCH-23,390 caused a significant decrease in hypoactivity in AB larvae during the dark phase, the same concentration did not cause any effect in 5D larvae.

Although these results show that movement regulation is conserved in zebrafish, confirmation that the DA signaling pathway is the modulator of the observed effects is needed including assessment of TH, dopamine, and metabolites levels.

## 8. Conclusions

The discussed studies provide supporting evidence that zebrafish under chemical challenges or genetic manipulation can be used to induce effects comparable to those observed in other animal models for translation to human dopaminergic system development and neurodegeneration. However, the lack of a standard protocol limits direct comparison among many of the studies and raises questions about their validity. Some of the studies that investigated the effects of neurotoxicants on zebrafish to induce PD-like characteristics started exposure to the chemicals during development of DA neurons and used only in situ hybridization to characterize DA neurons, without using quantitative methods to measure protein levels or levels of dopamine and its metabolites. Additionally, it is not clear whether these toxicants affect differentiation or survival of DA neurons; therefore, consideration of the neurotoxicant exposure regime should be carefully chosen and well detailed in the study. For example, exposure should be done after complete development of the DA system and BBB, if the goal of the study is to assess neurodegeneration of these neurons. In addition, some studies used *th* as a marker for DA neurons, while others used *th* and *dat*. As such, it is difficult to compare the results since *th* is a marker for all catecholaminergic neurons and *dat* is more specific for only DA neurons. Moreover, current genetic models most commonly used morpholino knockdown technologies. Additional transgenic models utilizing CRISPR-based techniques will enhance functional characterization of the key genetic targets. Moreover, observed transient effects of neurotoxicants on DA neurons highlights the potential of the zebrafish to be a model for studying neuronal regeneration.

## Figures and Tables

**Table 1:** Zebrafish genes involved in dopaminergic neuronal development.

Zebrafish Common Name	Zebrafish Gene Symbol	Human Gene Symbol	ZFIN ID (www.zfin.org)
glial cell derived neurotrophic factor a	*gdnfa*	*GDNF*	ZDB-GENE-010226-1
nuclear receptor subfamily 4, group A, member 2a	*nr4a2a*	*NR4A2*	ZDB-GENE-990415-184
nuclear receptor subfamily 4, group A, member 2b	*nr4a2b*	*NR4A2*	ZDB-GENE-040718-103
LIM homeobox transcription factor 1, beta b	*lmx1bb*	*LMX1B*	ZDB-GENE-050114-2
LIM homeobox transcription factor 1, beta a	*lmx1ba*	*LMX1B*	ZDB-GENE-050114-3
orthopedia homeobox a	*otpa*	*OTP*	ZDB-GENE-070216-1
orthopedia homeobox b	*otpb*	*OTP*	ZDB-GENE-990708-7
aristaless related homeobox a	*arxa (arx)*	*ARX*	ZDB-GENE-990415-15
ISL LIM homeobox 1	*isl1*	*ISL1*	ZDB-GENE-980526-112

## References

[1] Howe K., Clark M.D., Torroja C.F., Torrance J., Berthelot C., Muffato M., Collins J.E., Humphray S., McLaren K., Matthews L. (2013). The zebrafish reference genome sequence and its relationship to the human genome. Nature.

[2] Horzmann K., Freeman J. (2016). Zebrafish Get Connected: Investigating Neurotransmission Targets and Alterations in Chemical. Toxics.

[3] Sulzer D. (2007). Multiple hit hypotheses for dopamine neuron loss in Parkinson’s disease. Trends Neurosci..

[4] Rink E., Wullimann M.F. (2002). Development of the catecholaminergic system in the early zebrafish brain: An immunohistochemical study. Dev. Brain Res..

[5] Missale C., Nash S.R., Robinson S.W., Jaber M., Caron M.G. (1998). Dopamine receptors: From structure to function. Physiol. Rev..

[6] Benarroch E.E. (2013). Monoamine transporters: Structure, regulation, and clinical implications. Neurology.

[7] Torres G.E., Gainetdinov R.R., Caron M.G. (2003). Plasma membrane monoamine transporters: Structure, regulation and function. Nat. Rev. Neurosci..

[8] Sallinen V., Sundvik M., Reenilä I., Peitsaro N., Khrustalyov D., Anichtchik O., Toleikyte G., Kaslin J., Panula P. (2009). Hyperserotonergic phenotype after monoamine oxidase inhibition in larval zebrafish. J. Neurochem..

[9] Semenova S., Rozov S., Panula P. (2017). Distribution, properties, and inhibitor sensitivity of zebrafish catechol-O-methyl transferases (COMT). Biochem. Pharmacol..

[10] Lambert A.M., Bonkowsky J.L., Masino M.A. (2012). The conserved dopaminergic diencephalospinal tract mediates vertebrate locomotor development in zebrafish larvae. J. Neurosci..

[11] Guo S., Wilson S.W., Cooke S., Chitnis A.B., Driever W., Rosenthal A. (1999). Mutations in the zebrafish unmask shared regulatory pathways controlling the development of catecholaminergic neurons. Dev. Biol..

[12] Holzschuh J., Ryu S., Aberger F., Driever W. (2001). Dopamine transporter expression distinguishes dopaminergic neurons from other catecholaminergic neurons in the developing zebrafish embryo. Mech. Dev..

[13] Ren G., Li S., Zhong H., Lin S. (2013). Zebrafish tyrosine hydroxylase 2 gene encodes tryptophan hydroxylase. J. Biol. Chem..

[14] Schweitzer J., Löhr H., Filippi A., Driever W. (2012). Dopaminergic and noradrenergic circuit development in zebrafish. Dev. Neurobiol..

[15] Rink E., Wullimann M.F. (2001). The teleostean (zebrafish) dopaminergic system ascending to the subpallium (striatum) is located in the basal diencephalon (posterior tuberculum). Brain Res..

[16] Du Y., Guo Q., Shan M., Wu Y., Huang S., Zhao H., Hong H., Yang M., Yang X., Ren L. (2016). Spatial and Temporal Distribution of Dopaminergic Neurons during Development in Zebrafish. Front. Neuroanat..

[17] Lam C.S., Korzh V., Strahle U. (2005). Zebrafish embryos are susceptible to the dopaminergic neurotoxin MPTP. Eur. J. Neurosci..

[18] Sallinen V., Torkko V., Sundvik M., Reenilä I., Khrustalyov D., Kaslin J., Panula P. (2009). MPTP and MPP+ target specific aminergic cell populations in larval zebrafish. J. Neurochem..

[19] Tay T.L., Ronneberger O., Ryu S., Nitschke R., Driever W. (2011). Comprehensive catecholaminergic projectome analysis reveals single-neuron integration of zebrafish ascending and descending dopaminergic systems. Nat. Commun..

[20] Ryu S., Mahler J., Acampora D., Holzschuh J., Erhardt S., Omodei D., Simeone A., Driever W. (2007). Orthopedia homeodomain protein is essential for diencephalic dopaminergic neuron development. Curr. Biol..

[21] Boehmler W., Carr T., Thisse C., Thisse B., Canfield V.A., Levenson R. (2007). D4 Dopamine receptor genes of zebrafish and effects of the antipsychotic clozapine on larval swimming behaviour. Genes Brain Behav..

[22] Boehmler W., Obrecht-Pflumio S., Canfield V., Thisse C., Thisse B., Levenson R. (2004). Evolution and expression of D2 and D3 dopamine receptor genes in zebrafish. Dev. Dyn..

[23] Li P., Shah S., Huang L., Carr A.L., Gao Y., Thisse C., Thisse B., Li L. (2007). Cloning and spatial and temporal expression of the zebrafish dopamine D1 receptor. Dev. Dyn..

[24] Ryu S., Holzschuh J., Mahler J., Driever W. (2006). Genetic analysis of dopaminergic system development in zebrafish. J. Neural. Transm. Suppl..

[25] Holzschuh J., Hauptmann G., Driever W. (2003). Genetic analysis of the roles of Hh, FGF8, and nodal signaling during catecholaminergic system development in the zebrafish brain. J. Neurosci..

[26] Filippi A., Dürr K., Ryu S., Willaredt M., Holzschuh J., Driever W. (2007). Expression and function of nr4a2, lmx1b, and pitx3 in zebrafish dopaminergic and noradrenergic neuronal development. BMC Dev. Biol..

[27] Luo G.R., Chen Y., Li X.P., Liu T.X., Le W.D. (2008). Nr4a2 is essential for the differentiation of dopaminergic neurons during zebrafish embryogenesis. Mol. Cell. Neurosci..

[28] Jeong J.-Y., Einhorn Z., Mercurio S., Lee S., Lau B., Mione M., Wilson S.W., Guo S. (2006). Neurogenin1 is a determinant of zebrafish basal forebrain dopaminergic neurons and is regulated by the conserved zinc finger protein Tof/Fezl. Proc. Natl. Acad. Sci. USA.

[29] Filippi A., Jainok C., Driever W. (2012). Analysis of transcriptional codes for zebrafish dopaminergic neurons reveals essential functions of Arx and Isl1 in prethalamic dopaminergic neuron development. Dev. Biol..

[30] Del Giacco L., Sordino P., Pistocchi A., Andreakis N., Tarallo R., Di Benedetto B., Cotelli F. (2006). Differential regulation of the zebrafish orthopedia1 gene during fate determination of diencephalic neurons. BMC Dev. Biol..

[31] Wong C.E.D., Hua K., Monis S., Saxena V., Norazit A., Noor S.M., Ekker M. (2020). Gdnf affects early diencephalic dopaminergic neuron development through regulation of differentiation-associated transcription factors in zebrafish. J. Neurochem..

[32] Filippi A., Mueller T., Driever W. (2014). vglut2 and gad expression reveal distinct patterns of dual GABAergic versus glutamatergic cotransmitter phenotypes of dopaminergic and noradrenergic neurons in the zebrafish brain. J. Comp. Neurol..

[33] Tieu K. (2011). A guide to neurotoxic animal models of Parkinson’s disease. Cold Spring Harb. Perspect. Med..

[34] Kopin I.J. (1992). Features of the dopaminergic neurotoxin MPTP. Ann. N. Y. Acad. Sci..

[35] Bretaud S., Lee S., Guo S. (2004). Sensitivity of zebrafish to environmental toxins implicated in Parkinson’s disease. Neurotoxicol. Teratol..

[36] McKinley E.T., Baranowski T.C., Blavo D.O., Cato C., Doan T.N., Rubinstein A.L. (2005). Neuroprotection of MPTP-induced toxicity in zebrafish dopaminergic neurons. Brain Res. Mol. Brain Res..

[37] Wen L., Wei W., Gu W., Huang P., Ren X., Zhang Z., Zhu Z., Lin S., Zhang B. (2008). Visualization of monoaminergic neurons and neurotoxicity of MPTP in live transgenic zebrafish. Dev. Biol..

[38] Xi Y., Yu M., Godoy R., Hatch G., Poitras L., Ekker M. (2011). Transgenic zebrafish expressing green fluorescent protein in dopaminergic neurons of the ventral diencephalon. Dev. Dyn..

[39] de Souza Lima A.C.M., de Alvarenga K.A.F., Codo B.C., Sacramento E.K., Rosa D.V.F., Souza R.P., Romano-Silva M.A., Souza B.R. (2020). Impairment of motor but not anxiety-like behavior caused by the increase of dopamine during development is sustained in zebrafish larvae at later stages. Int. J. Dev. Neurosci..

[40] Sarath Babu N., Murthy C.L.N., Kakara S., Sharma R., Brahmendra Swamy C.V., Idris M.M. (2016). 1-Methyl-4-phenyl-1,2,3,6-tetrahydropyridine induced Parkinson’s disease in zebrafish. Proteomics.

[41] Anichtchik O.V., Kaslin J., Peitsaro N., Scheinin M., Panula P. (2004). Neurochemical and behavioural changes in zebrafish Danio rerio after systemic administration of 6-hydroxydopamine and 1-methyl-4-phenyl-1,2,3,6-tetrahydropyridine. J. Neurochem..

[42] Tanner C.M., Kamel F., Ross G.W., Hoppin J.A., Goldman S.M., Korell M., Marras C., Bhudhikanok G.S., Kasten M., Chade A.R. (2011). Rotenone, paraquat, and Parkinson’s disease. Environ. Health Perspect..

[43] Dauer W., Przedborski S. (2003). Parkinson’s disease: Mechanisms and models. Neuron.

[44] Nellore J., Nandita P. (2015). Paraquat exposure induces behavioral deficits in larval zebrafish during the window of dopamine neurogenesis. Toxicol. Rep..

[45] Wang Q., Liu S., Hu D., Wang Z., Wang L., Wu T., Wu Z., Mohan C., Peng A. (2016). Identification of apoptosis and macrophage migration events in paraquat-induced oxidative stress using a zebrafish model. Life Sci..

[46] Kalyn M., Hua K., Noor S.M., Wong C.E.D., Ekker M. (2020). Comprehensive analysis of neurotoxin-induced ablation of dopaminergic neurons in Zebrafish Larvae. Biomedicines.

[47] Bortolotto J.W., Cognato G.P., Christoff R.R., Roesler L.N., Leite C.E., Kist L.W., Bogo M.R., Vianna M.R., Bonan C.D. (2014). Long-term exposure to paraquat alters behavioral parameters and dopamine levels in adult zebrafish (Danio rerio). Zebrafish.

[48] Nunes M.E., Müller T.E., Braga M.M., Fontana B.D., Quadros V.A., Marins A., Rodrigues C., Menezes C., Rosemberg D.B., Loro V.L. (2017). Chronic Treatment with Paraquat Induces Brain Injury, Changes in Antioxidant Defenses System, and Modulates Behavioral Functions in Zebrafish. Mol. Neurobiol..

[49] Brooks A.I., Chadwick C.A., Gelbard H.A., Cory-Slechta D.A., Federoff H.J. (1999). Paraquat elicited neurobehavioral syndrome caused by dopaminergic neuron loss. Brain Res..

[50] Ren J.-P., Zhao Y.-W., Sun X.-J. (2009). Toxic influence of chronic oral administration of paraquat on nigrostriatal dopaminergic neurons in C57BL/6 mice. Chin. Med. J..

[51] Vaz R.L., Outeiro T.F., Ferreira J.J. (2018). Zebrafish as an animal model for drug discovery in Parkinson’s disease and other movement disorders: A systematic review. Front. Neurol..

[52] Feng C.-W., Wen Z.-H., Huang S.-Y., Hung H.-C., Chen C.-H., Yang S.-N., Chen N.-F., Wang H.-M., Hsiao C.-D., Chen W.-F. (2014). Effects of 6-hydroxydopamine exposure on motor activity and biochemical expression in zebrafish (Danio rerio) larvae. Zebrafish.

[53] Vijayanathan Y., Lim F.T., Lim S.M., Long C.M., Tan M.P., Majeed A.B.A., Ramasamy K. (2017). 6-OHDA-Lesioned Adult Zebrafish as a Useful Parkinson’s Disease Model for Dopaminergic Neuroregeneration. Neurotox. Res..

[54] Sager J.J., Bai Q., Burton E.A. (2010). Transgenic zebrafish models of neurodegenerative diseases. Brain Struct. Funct..

[55] Kim R.H., Smith P.D., Aleyasin H., Hayley S., Mount M.P., Pownall S., Wakeham A., You-Ten A.J., Kalia S.K., Horne P. (2005). Hypersensitivity of DJ-1-deficient mice to 1-methyl-4-phenyl-1,2,3,6- tetrahydropyrindine (MPTP) and oxidative stress. Proc. Natl. Acad. Sci. USA.

[56] Bretaud S., Allen C., Ingham P.W., Bandmann O. (2007). p53-dependent neuronal cell death in a DJ-1-deficient zebrafish model of Parkinson’s disease. J. Neurochem..

[57] Xi Y., Ryan J., Noble S., Yu M., Yilbas A.E., Ekker M. (2010). Impaired dopaminergic neuron development and locomotor function in zebrafish with loss of pink1 function. Eur. J. Neurosci..

[58] Sallinen V., Kolehmainen J., Priyadarshini M., Toleikyte G., Chen Y.-C., Panula P. (2010). Dopaminergic cell damage and vulnerability to MPTP in Pink1 knockdown zebrafish. Neurobiol. Dis..

[59] Kitada T., Asakawa S., Hattori N., Matsumine H., Yamamura Y., Minoshima S., Yokochi M., Mizuno Y., Shimizu N. (1998). Mutations in the parkin gene cause autosomal recessive juvenile parkinsonism. Nature.

[60] Flinn L., Mortiboys H., Volkmann K., Köster R.W., Ingham P.W., Bandmann O. (2009). Complex I deficiency and dopaminergic neuronal cell loss in parkin-deficient zebrafish (Danio rerio). Brain.

[61] Irons T.D., Kelly P.E., Hunter D.L., MacPhail R.C., Padilla S. (2013). Acute administration of dopaminergic drugs has differential effects on locomotion in larval zebrafish. Pharmacol. Biochem. Behav..

[62] Farrell T.C., Cario C.L., Milanese C., Vogt A., Jeong J.-H., Burton E.A. (2011). Evaluation of spontaneous propulsive movement as a screening tool to detect rescue of Parkinsonism phenotypes in zebrafish models. Neurobiol. Dis..

[63] Oliveri A.N., Levin E.D. (2019). Dopamine D 1 and D 2 receptor antagonism during development alters later behavior in zebrafish. Behav. Brain Res..

